# Real-life comparison of the viewing angle and the image quality of two commonly used viewing systems for vitreoretinal surgery

**DOI:** 10.3906/sag-1910-11

**Published:** 2020-06-23

**Authors:** Kemal BAYRAKÇEKEN, Ahmet Murad HONDUR, Tuba ATALAY, Yavuz Kemal ARIBAS

**Affiliations:** 1 Department of Ophthalmology, Gazi University, Ankara Turkey

**Keywords:** Image quality, viewing angle, vitreoretinal surgery, wide angle viewing system.

## Abstract

**Background/aim:**

To compare the clinical use, image quality and viewing angle of a commonly used contact wide angle viewing (WAV) system (Advanced Visual Instruments (AVI) Panoramic Imaging Systems, NY, USA) with a commonly used noncontact WAV system (Leica RUV800, Leica Microsystems, Switzerland).

**Materials and methods:**

Images of 42 consecutively operated eyes were obtained with both systems at the same surgical stages and were compared for image quality using the Imatest Master 4.5.13 (Imatest LLC, Boulder, USA) software. The viewing angles of the images were calculated using the optic disc sizes measured from the OCT and infrared fundus images. The 68-degree AVI lens was compared with the 90-dioptre (D) Leica RUV800 lens, while the 130-degree AVI lens was compared with the 132-D Leica RUV800 lens. The surgical assistants were asked to grade the difficulty of holding the lens in place from 1 to 10, 1 being the easiest and 10 being the most difficult.

**Results:**

The contact system provided wider viewing angles with higher quality compared to the noncontact system both under fluid and air media. The difference was clinically significant in eyes with impaired corneal clarity, very high myopia, or small pupil. The difficulty of holding the lens in place ranged from 4 to 7, and decreased gradually with practice.

**Conclusions:**

Both WAV systems provided high image quality and adequate viewing angles in most cases. However, the contact system appeared to provide a superior image quality and/or a wider viewing angle in more challenging situations. The difficulty of holding the contact lens in place was found to be moderate.

## 1. Introduction

A wide field of surgical view with high image quality is critical for the safety and efficacy of vitreoretinal surgical procedures. Currently, the two major approaches for vitreoretinal viewing are the contact and the noncontact wide angle viewing (WAV) systems. Both contact and noncontact systems offer potential advantages but have theoretical limitations and drawbacks as well [1–3].

Various reports have presented the use of separate systems theoretically [4–10]. Although the specifications of each system are usually revealed in the brochures and reports, the description is often different among the manufactures, and therefore not directly comparable. The optical design appears to be the key industrial proprietary secret in each system, hence is not always or completely open to surgeons for comparison of the specifications. 

However, although it has not been performed to the best of our knowledge, a clinical comparison may be performed. In the present study, we aimed to compare the clinical use, image quality and viewing angle of a commonly used contact WAV system (68 and 130 Degree lenses, Advanced Visual Instruments (AVI) Panoramic Imaging Systems, New York, USA) with a commonly used noncontact WAV system (90 Dioptres (D) and 132 D XL lenses, Leica RUV800 Panoramic Viewing System, Leica Microsystems, Switzerland).

## 2. Materials and methods

The study protocol was approved (with a waiver of consent) by the Institutional Review Board of Gazi University Medical School, and the tenets of the Declaration of Helsinki were followed. Forty-two eyes of consecutive 42 patients who underwent vitreoretinal surgery were included in this study. All patients underwent a complete preoperative ocular evaluation including systemic disease history, refraction, measurement of the best-corrected visual acuity, slit-lamp biomicroscopy, intraocular pressure with a noncontact tonometry and funduscopy.

All of the vitreoretinal surgeries were performed by a single surgeon under the same surgical microscope (Leica M844, Leica Microsystems, Switzerland) in order to avoid optical and viewing differences that could arise from microscope differences. 

The surgeon was assisted by senior residents of the retina service, who were asked to grade the difficulty of holding the lens in place from 1 to 10, 1 being the easiest and 10 being the most difficult. 

The images were obtained consecutively with the contact WAV system (68 and 130 Degree AVI lenses) and the noncontact WAV system (90 D and 132 D XL lenses, Leica RUV800) at the same surgical stages in order to make the comparison possible. For this aim, the focus and magnification of the surgical microscope were adjusted to obtain the clearest image quality and the biggest possible image size fitting into the picture frame. 

The optic disc diameters of the operated eyes were calculated using the optical coherence tomography (OCT) images and infrared fundus images. Then, using the optic disc diameter the image sizes (viewing angles) were calculated in degrees, converting 250 micrometres (mm) to 1 degree (o). 

The quality (resolution) of images was evaluated with the Imatest Master 4.5.13 (Imatest LLC, Boulder, USA) image quality analysis program. The spatial frequency response (SFR) module was used to assess the image sharpness (clarity), which is widely accepted as the most important parameter in evaluating image quality [11]. In this module, Modulation Transfer Function 50 (MTF50) value (in cycles/pixel) for each image, which is widely accepted as the gold standard in the evaluation of image quality, was recorded. MTF50 refers to MTF that is 50% of its low frequency value (MTF50) or 50% of its peak value (MTF50P) [11]. 

The images acquired with the 130-degree AVI contact lens were compared with those acquired with the 132 D XL noncontact Leica RUV800 lens (the wide angle lenses of each system), while the images acquired with the 68-degree AVI contact lens were compared with the 90 D noncontact Leica RUV800 lens (the posterior pole lenses of each system).

The comparison of the corresponding images obtained with the 2 systems was performed under same optic conditions; images obtained under fluid were compared with ones obtained under fluid, and similarly images obtained under air were compared with ones obtained under air with the other WAV system. Four eyes of 4 patients were excluded from the comparison due to unsatisfactory view of the fundus with the noncontact system.

Statistical analyses were performed with the SPSS 22.0 package program (IBM Corp., Armonk, NY, USA). The t-test was used for the comparison of groups. P-values ​​less than 0.05 were considered statistically significant in the study.

## 3. Results

Forty-two patients (24 men and 18 women) were included in this study. Mean patient age was 49.7 ± 18.8 years (range: 4.5–79 years). The ocular and surgical features of the cases are outlined in Table 1.

**Table 1:** Ocular and surgical features of the cases

Eye	Right			Left		
	20 (47.6%)			22 (52.4%)		
Lens status	Phakic		Pseudophakic		Aphakic	
	12 (28.6%)		26 (61.9%)		4 (9.5%)	
Pupil Size	≤ 5 mm		> 5 mm		Mean pupil size: 6.83 ± 1.31mm (range: 4-9.5mm)	
	7 (16.7%)		35 (83.3%)			
Indication for Surgery	RD	PDR/TRD	ERM	MH	IOFB	Miscellaneous
	14 (33.3%)	7 (16.7%)	10 (23.8%)	5 (11.9%)	1 (2.4%)	5 (11.9%)

RD: Retinal detachment, PDR: Proliferative diabetic retinopathy, TRD: Tractional retinal detachment, ERM: Epiretinal membrane, MH: Macular hole, IOFB: Intraocular foreign body.

**Figure 1:**
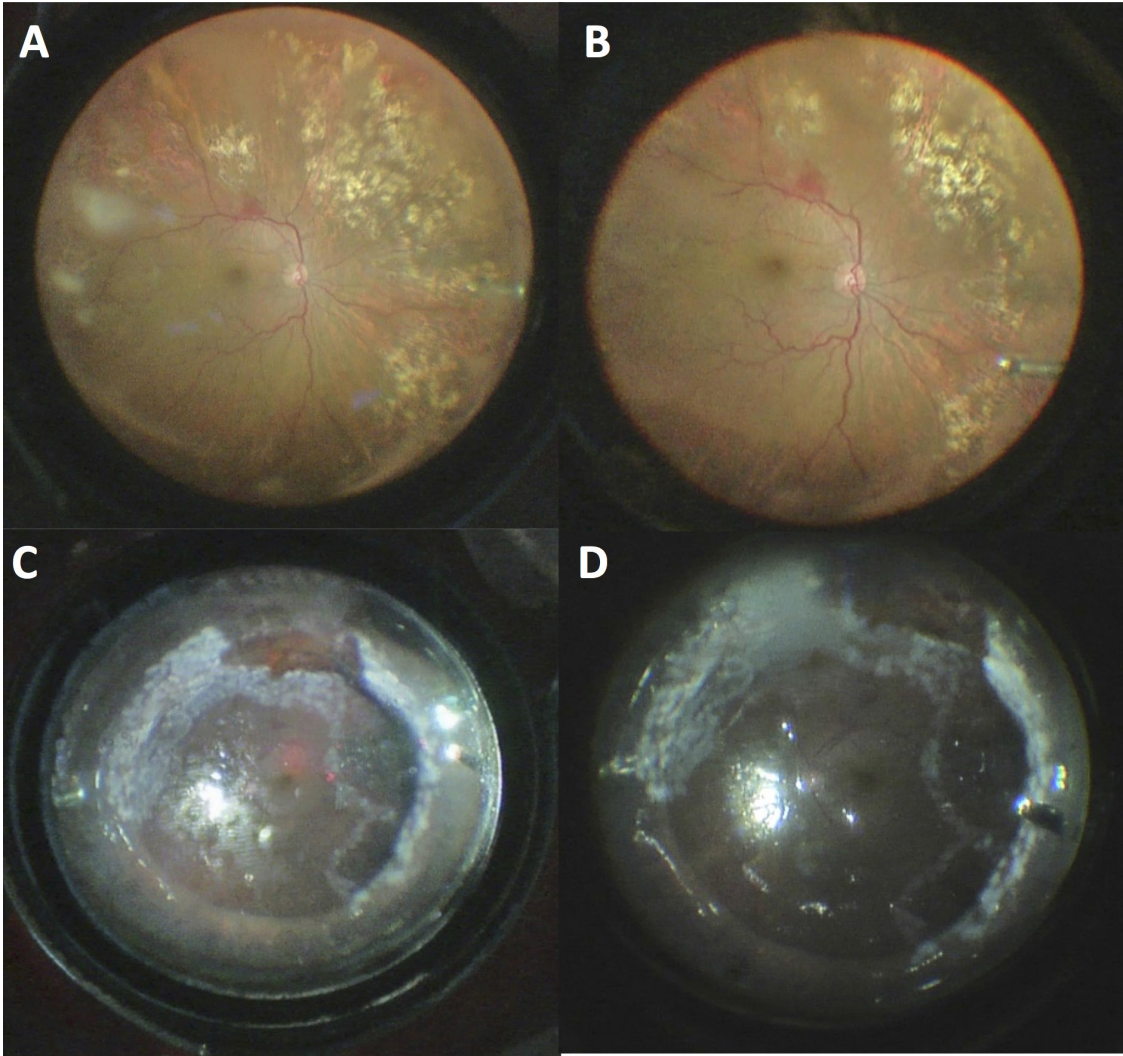
A wider viewing angle and better visualization of the surgical field are noted with the AVI 130-degree lens (A, 110 degrees) compared to the Leica RUV800 XL lens (B, 90 degrees) under fluid in the images from the same step of surgery. Similarly, a wider angle and better visualization of retina with the AVI 130-degree lens (C, 165 degrees) compared to the Leica RUV800 XL lens (D, 110 degrees) are noted under air in images from a different surgery.

**Figure 2:**
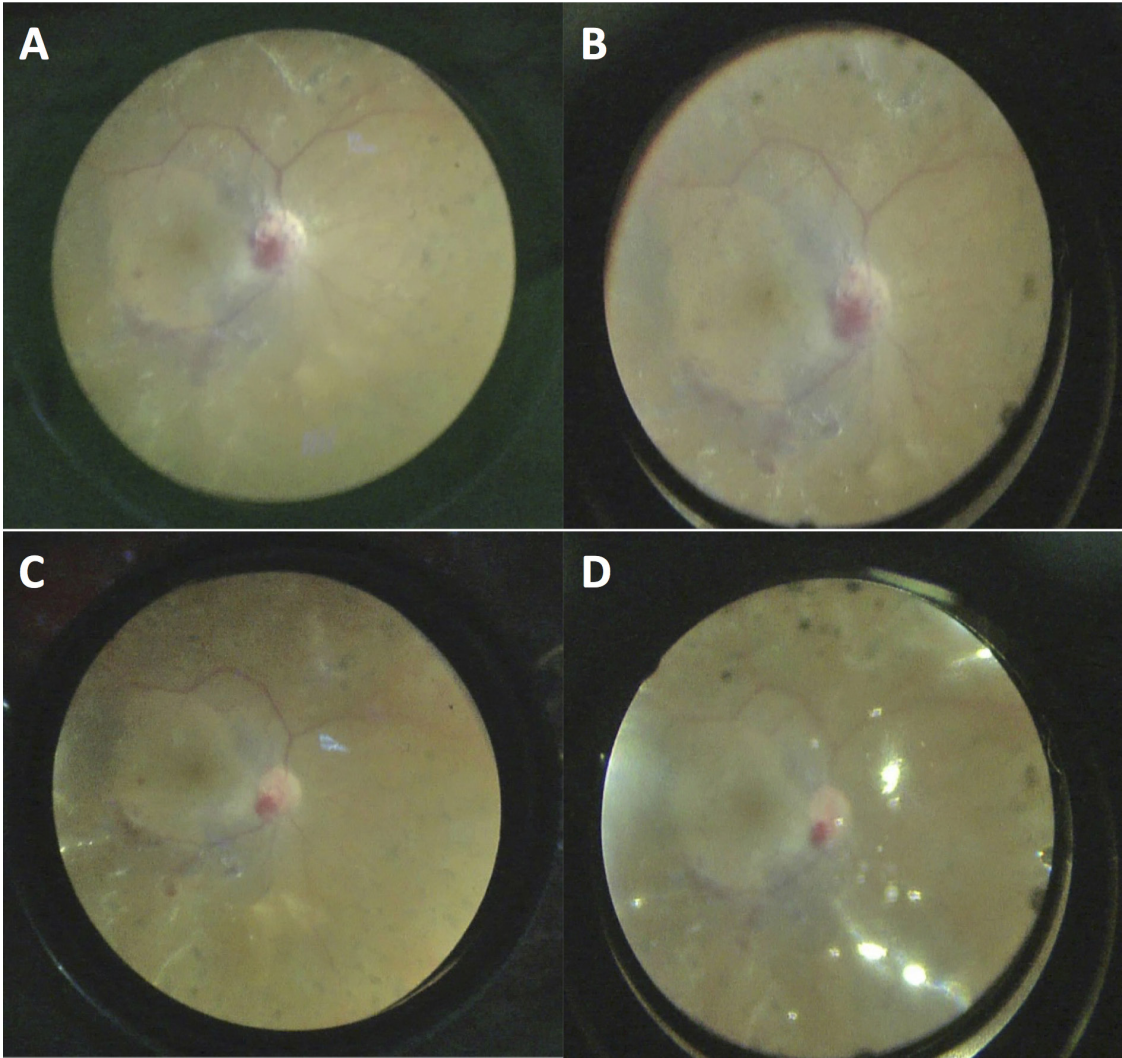
The AVI 68-degree lens (A, 70 degrees) provided a wider viewing angle and better visualization under fluid compared to the Leica RUV800 90 D lens (B, 50 degrees) in the images taken at the same step of surgery. Similarly, a wider angle and better visualization of retina with the AVI 68-degree lens (C, 110 degrees) compared to the Leica RUV800 XL lens (D, 75 degrees) are noted under air in images from the same surgery.

The contact WAV system (Advanced Visual Instruments Panoramic Imaging System) lenses provided wider viewing angles with higher quality (sharpness) compared to the corresponding noncontact WAV system (Leica RUV800) lenses (Table 2), both under fluid and air media (Figures 1–2).

**Table 2:** Comparison of Image Quality and Viewing Angle of the Two Wide-Angle Viewing Systems under Different Optic Media

Image Quality(cycles/pixel)		AVI 130o	L-RUV 132 D		AVI 68o	L-RUV 90 D	
	Under Fluid	0.03 ± 0.02	0.01 ± 0.01	p= 0.01(n= 21)	0.06 ± 0.11	0.01 ± 0.01	p= 0.01(n= 36)
	Under Air	0.07 ± 0.17	0.01 ± 0.01	p= 0.01(n= 21)	0.10 ± 0.17	0.01 ± 0.01	p= 0.01(n= 36)
Viewing Angle(Degrees)		AVI 130o	L-RUV 132 D		AVI 68o	L-RUV 90 D	
	Theoretical*	130o	124o		68o	90˚	
	Under Fluid	120.8o ± 8.3o[92.9±6.4%]	97.9o ± 10.6o[79.0±8.5%]	p=0.01(n= 21)	85.3o ± 10.2o[125.4±15.0%]	64.6o ± 9.6o[71.8±10.7%]	p= 0.01(n= 36)
	Under Air	152.6o ± 23.8o[117.4±18.3%]	113.7o ± 15.6o[91.7±12.6%]	p= 0.01(n= 21)	105.6o ± 12.0o[155.3±17.5%]	83.8o ± 12.2o[93.1±13.3%]	p= 0.01(n= 36)

o: Degrees, L-RUV: Leica RUV800, D: Diopters, n: Number of cases, *: Under fluid medium, Values in brackets: Viewing angles as percentage of the theoretical viewing angle.

**Figure 3:**
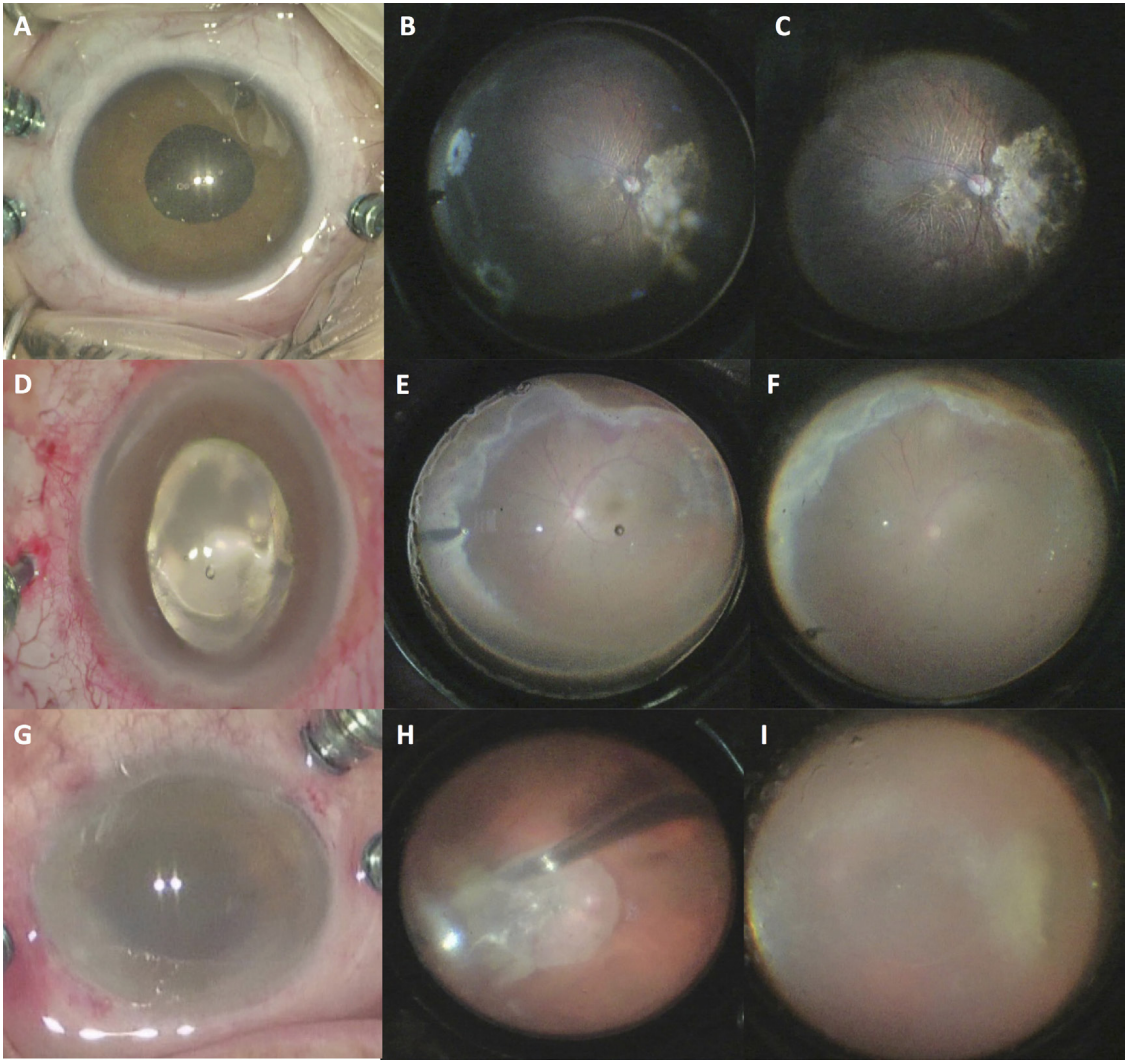
In an eye with a pupil size of 4.5 mm under fluid medium the AVI 130-degree lens provided a viewing angle of 100 degrees (A), which made the temporally located 2 peripheral tears visible (B). With the RUV800 XL lens the viewing angle was 80 degrees, and the tears on the temporal side were not visible under fluid medium without scleral indentation (C). In another eye with traumatic corneal edema (D) and retinal detachment, under fluid medium the AVI 130-degree lens (E) provided a wider viewing angle and a much clear and detailed visualization of the fundus than the RUV800 XL lens (F). In another eye with a dropped nucleus and cataract surgery induced corneal edema (G), under fluid medium the AVI 68-degree lens (H) provided a wider viewing angle and a much clear and detailed visualization of the fundus than the RUV800 90 D lens (I).

Compared to the nominal viewing angles described by the manufacturer, only the AVI 68-degree lens provided a wider mean angle than its nominal 68 degrees. The mean viewing angle of the AVI 130-degree lens was about 10 degrees narrower than the nominal 130 degrees under fluid medium. On the other hand, the mean viewing angles provided by the noncontact WAV system lenses were much narrower than their nominal values (Table 2, Figures 1, 2 and 3A-C).

The reported mean grade of difficulty for holding the lens in place by surgical assistants was 6 (range 5 to 7) for the first 3 cases assisted, and 4.5 (range 4 to 5) after assisting 10 cases. 

Although the contact lenses provided a higher quality and wider viewing angles, the image quality and viewing angles of the noncontact WAV system (Leica RUV800) were satisfactory for safe and efficient surgery in most cases. But, in 4 eyes which were excluded from the image comparison due to unsatisfactory quality of view of the fundus with the noncontact system, the surgeries were performed exclusively with the contact lenses. These cases were an eye with very high myopia (axial length of 36 mm), a retinal detachment in an eye with a keratoconus scar, a traumatic retinal detachment in an eye with corneal edema (Figure 3D-F), a dropped nucleus in an eye with cataract surgery induced corneal edema (Figure 3G-I), and in another case, the surgery was performed exclusively with the contact AVI 130-degree lens as the viewing angle was unsatisfactory with the RUV800 132 D XL lens due to a small pupil size.

## 4. Discussion

The ideal retinal imaging system should offer a high-quality image of the retina with a wide viewing angle, both of which are important for a safe and efficient surgery. A better surgical view is also a key factor for development of new surgical manoeuvres and techniques [9].

In this study, we compared the image quality and viewing angle of a commonly used contact WAV system (Advanced Visual Instruments Panoramic Imaging Systems) with another commonly used noncontact WAV system (Leica RUV800). Although, the potential advantages, limitations, and drawbacks are anticipated theoretically or have been experienced subjectively, there has not been any quantitative real life comparison of the 2 major viewing approaches (contact versus noncontact) in vitreoretinal surgery. In addition, although variations in the theoretical viewing angle under different surgical conditions have been anticipated [2], this has remained an unstudied subject.

For analysis of image quality, the MTF50 values calculated with the SFR module of the Imatest Master 4.5.13 (Imatest LLC, Boulder, USA) image quality analysis software was used. This approach provides an objective tool for comparison independent of observer bias, hence is accepted as a proper indicator of image quality (sharpness or resolution). It is also accepted to be superior to traditional resolution measurements which involve visualization of an image of a bar pattern (often the USAF 1951 chart). The traditional measurement is also known to be strongly dependent on observer bias, and hence a poor indicator of image sharpness [11].

In the present study, the contact AVI lenses provided higher quality views (sharper or higher resolution images) of the retina compared to corresponding the noncontact Leica RUV800 lenses (Table 2, Figures 1–2). Theoretically, this difference mostly rises from scattering and reflection of light between cornea and the noncontact lens system, which does not happen in the contact system as the lens is in apposition with the cornea. Contact WAV systems also eliminate the corneal aberrations, which also contributes to the better image quality [1]. On the other hand, the corneal shape may occasionally be distorted by the assistant with contact lenses, which may distort the image transiently. 

In the majority of cases the difference in the image quality of the two systems was not clinically significant, and did not impede a safe and effective surgery with the Leica RUV800 noncontact WAV lenses. However, in cases with corneal disturbances such as edema, haze or scar, we experienced that the difference became critical for effective surgical viewing, and with noncontact lenses accomplishing vitreoretinal surgery without artificial keratoprosthetics and subsequent keratoplasty would become impossible. This was experienced in three cases; a retinal detachment in an eye with a keratoconus scar, a traumatic retinal detachment in an eye with corneal edema, and a dropped nucleus in an eye with cataract surgery induced corneal edema (Figures 3D–F). Although infrequent, performing vitrectomy with same session keratoplasty makes the surgery more complex, requires the presence of a corneal surgeon and the availability of a donor corneal graft. Combined surgery may also increase intraocular inflammation compared to vitrectomy alone, and may be a negative factor for corneal graft survival.

In another case with very high myopia, the focusing of the noncontact system was not adequate to achieve a clear view of the retina under air in this study. When the vitreous cavity is filled with air, the refractive power of the eye focuses the imaginary image of the retina at about 2 mm in front of the cornea in an emmetropic eye [9]. In a very high myopic eye (with much greater refractive power), this distance decreases to about 1 mm or less, and hence the noncontact system cannot capture the image without inadvertent touch to the corneal surface. While using the contact lenses, the wide range of focusing, which is adjusted by changing the distance between the microscope and the lens, easily permits a clear view of the fundus even in very high myopic eyes.

The width of the view is the other important factor for a WAV system, because failures in vitreoretinal surgery often result from missed or residual pathologies in the vitreous base [7]. The viewing angles were also superior with the AVI lenses compared to their RUV800 counterparts (Table 2, Figures 1–3). Theoretically, this results from the (contact WAV) lens being placed at the nearest distance to the cornea, while there has to be a distance between the lens and cornea in the noncontact WAV systems. Though, the noncontact RUV800 lenses also provided adequate field of view in the majority of cases. In only a single case, the viewing angle was unsatisfactory with the RUV800 132 D XL lens due to a small pupil size, and the surgery was performed exclusively with the contact AVI 130-degree lens. This avoided the use of iris retraction hooks, which can lead to subsequent pupillary irregularities, increased intraocular inflammation, and lenticular damage in phakic eyes. Avoiding the use of iris retraction hooks also shortens the surgical duration.

The wider viewing angles with the AVI lenses compared to their RUV800 counterparts confirms the results of theoretical calculations and experimental studies. However, an interesting result in this clinical study was that the theoretically expected image widths were not met with the 2 noncontact lenses (Leica RUV800 90 D and 132 D XL) and 1 contact (AVI 130-degree) lens under fluid medium (Table 2). This most probably arises from the fact that the real-life clinical conditions do not meet the theoretical ideal conditions under which the nominal viewing angles are calculated. A smaller corneal diameter, a deeper anterior chamber and a smaller pupil size (than the ideal theoretical calculation), peripheral corneal haze (such as arcus senilis), the anterior capsulorhexis width and peripheral posterior capsule opacities in pseudophakic patients may all lead to a narrower field of view. The majority (61.9%) of the cases in this study were pseudophakic. Hence, the anterior capsulorhexis dimension, which is ideally about 5.5 mm, and peripheral posterior capsule opacities, which ideally cannot be removed beyond the central 5.5 mm probably resulted the real-life viewing angles to be narrower than the nominal values. Moreover, the noncontact lenses appeared to be affected more than the corresponding contact lenses (Table 2).

The evolution of vitrectomy towards smaller gauge (G) surgery puts further importance to the viewing angles of the WAV systems. As the conjunctiva is not opened with the use of trocars, visualization of the peripheral retina with scleral indentation is limited, particularly on the nasal side [12]. In addition, the reduced rigidity of the 25 and 27 G instruments also limits the visualization of the peripheral retina with noncontact WAV systems, as these systems require tilting of the globe towards the part intended to be visualized. With 20 or 23 G instruments, the rigidity of the instruments endures the tilting manoeuvres while the reduced endurance of smaller G, particularly 27 G, instruments may limit these manoeuvres. On the other hand, with contact WAV systems such manoeuvres are not required, and the surgeon uses the port of entry as a fulcrum without tilting the globe. Additionally, the assistant holding the (contact WAV) lens can further increase the visualization of the periphery by slightly moving the lens on cornea to the opposite direction; if the surgeon wants to see the nasal periphery the assistant would slightly slide the lens temporally on the cornea. With use of the AVI contact WAV system, a recent study has reported effective shaving of the vitreous base without scleral indentation in small-gauge (23 to 27 gauge) vitrectomy for retinal detachment. This study included complex retinal detachments and the single surgery reattachment rate was 95%, with a final reattachment rate of 99% [13].

The only reported disadvantage of contact WAV systems is the need for an assistant to hold the lens on the cornea [3,7,10,14]. Holding the lens was easily accomplished by senior residents of the retina service, who reported the mean grade of difficulty for holding the lens in place as moderate (4.5–6/10) and experienced a fast learning curve (in about 10 cases). In addition, lens retaining rings are available and other methods of lens self-stabilization have been described [7,10,14]. On the other hand, an inconvenience of the noncontact WAV systems is occasional fogging or condensation on the lens, which may result from suboptimal draping, a deep-set eye, fluid accumulation at the medial canthus, or cold room temperature. This inconvenience may be overcome by additional precautions such as use of antifog solutions, careful draping, optimal positioning of the patient’s head [2,15].

In conclusion, both of the tested WAV systems (the contact AVI lenses and the noncontact Leica RUV800 lenses) provided high image quality and adequate viewing angles for safe and effective vitreoretinal surgery in majority of cases. However, the real-life viewing angles were found to be narrower than the nominal values, particularly for noncontact lenses. In addition, the contact lenses appeared to be superior in more challenging cases, such as eyes with corneal edema and scar, small pupil, and high myopia. The contact lenses also provide the opportunity of performing surgery in a stationary position without rotating the globe to visualize the periphery, which may be important in accomplishing small gauge, particularly 27-gauge surgery. The only disadvantage of the contact lenses, which is the need for an assistant to hold the lens, was not found to be a major difficulty.

## Conflict of Interest

The authors report no conflict of interest, or any financial interest in any of the materials and methods used or described. The authors are responsible for the content and writing of the paper.
